# Phylogenetic lineage of GII.17 norovirus identified among children in South-South, Nigeria

**DOI:** 10.1186/s13104-020-05185-0

**Published:** 2020-07-22

**Authors:** Favour Osazuwa, Hailey Seth Grobler, William Johnson

**Affiliations:** 1 MDS Molecular Services Sub-Saharan African Centre, Abuja, Nigeria; 2 MDS Molecular Services, Johannesburg, South Africa; 3 Department of Medical Laboratory Sciences, University of Benin, Benin City, Nigeria

**Keywords:** Norovirus, GII.17, Children, RT-PCR

## Abstract

**Objectives:**

Norovirus is a major cause of diarrhea among children worldwide. This present report highlight’s the genetic homology patterns of GII.17 noroviruses detected among children under-5 years of age with diarrhea in the South-South, region of Nigeria. Stool specimens were collected from 300 children with diarrhea and analyzed for norovirus using conventional reverse transcriptase-Polymerase Chain Reaction. Sequencing of the capsid region was performed to genotype the strains

**Results:**

36/300 (12.0%) of patients were positive for norovirus by RT-PCR. 7/36 (19.4%) (5 GI.3 and 2 GI.5) were GI others where typed to be GII. All GII.17 norovirus identified in this study, 3/29 (10.3%) where typed to belong to the recently discovered GII.17 Kawasaki strain. This study report for the first time the detection of norovirus GII.17 Kawasaki strain in South-South, region of Nigeria.

## Introduction

Norovirus has been identified to constitute a key biological cause of gastroenteritis worldwide [1]. norovirus cause an estimated 1.1 million hospitalizations and up to 218,000 deaths among children less than 5 years annually [1]. norovirus exhibits high genetic diversity, currently at least ten genogroups exists, over 8 and 35 genotypes of GI and GII are known [2].

Though it is established that GII.4 are predominant in most cases of diarrhea illnesses [3], it is noteworthy to report other genotypes whose genetic characteristics are of public health concern. Incidence of hitherto rare GII.17 noroviruses has been reported to be increasing [4]. GII.17 norovirus genotype has now been documented to be identified to be the predominant cause of norovirus outbreaks in certain parts of the world [4]. GII.17 has over 5 variants, continuous viral evolution as a result of recombination events [5], is fueling the generation of new more virulent genotypic variants. This study presents the prevalence and phylogenetic characteristics of GII.17 identified among under 5 year old children with diarrhea in the South-South region, Nigeria.

## Main text

### Study area and study population

This cross-sectional study was conducted from March, 2018 to February, 2019. A total of 300 children with diarrhea from a pool of 1856, attending four secondary health facilities (Central Hospital, warri, Central Hospital, Benin, Primary Health Centre Pessu and Federal Medical Centre, Yenagoa) in Delta, Edo and Bayelsa States, Niger-Delta region, Nigeria were randomly included. Children with at least 3 episodes of diarrhea- with an onset of 1–7 days whose parents or guardians consented for their ward/children to participate were included in this study.

## Methods

### Sample collection and processing

Stool specimens were collected into clean universal containers. Supernatant obtained from stool suspension of 50% in 1 ml sterile phosphate buffered saline were stored at − 20 °C for RT-PCR analysis of norovirus.

#### RNA extraction

RNA extraction was carried out using AccuPrep® Viral RNA Extraction Kit (Bioneer, Daejon South Korea). Stepwise protocols as recommended by the manufacturer's instructions were followed.

#### cDNA synthesis

cDNA synthesis was carried out on a 20 µl reverse transcription reaction of 1.0 µg of extracted RNA on 0.2 ml tubes of Accupower Cycle script RT Premix (Bioneer Corporation, South Korea). Stepwise protocols as recommended by the manufacturer's instructions were followed.

### Polymerase chain reaction

The cDNA generated was then amplified by PCR in a 45 µl reaction mixture as described in a previous study [6]. Specific primers (G1SKRCAACCCARCCATTRTACA) and G1FFN (GGAGATCGCAATCTCCTGCCC) were used for GI genotyping, while for genotyping GII noroviruses, primers GIIFBN (TGGGAGGGCGATCGCAATCT) and GIISKR (CCRCCNGCATRHCCRTTRTACAT), respectively, were used in an RT-PCR analysis. The products were visualized on UV illuminator and photographed using Polaroid camera [7]. The RT-PCR used is a very sensitive method, it can detect as few as 5 × 10^6^ copies per gram of stool sample. U-TaQ DNA polymerase (SBS genetech, Beijing, China), a high fidelity thermostable enzyme that can withstand prolonged incubation at high temperature up to 95 °C without significant loss of activity was used for this RT-PCR protocol.

#### Norovirus sequencing

The amplicons from the partial gene regions of the viral capsid genes were purified using QIAquick PCR purification kit (Qiagen Inc., Valencia, CA). Nucleotide sequencing was done using Big Dye ® Terminator v 3.1 Cycle sequencing kit (Applied Biosystems, Carlsbad, CA) on 3130 DNA genetic analyzer (Applied Biosystems, Carlsbad, CA), Sequences were edited using sequencher® Version 5.4.6 DNA sequence analysis software (Gene codes Corporation, Ann Arbor, MI, USA). Norovirus genotypes were determined by comparison of corresponding sequences of norovirus strains using the online norovirus genotyping tool version 1.0. available at (www.rivm.nl/mpf/norovirus/typing tool).

#### Phylogenetic analysis

For confirmation of genotyping, nucleotide sequences obtained were aligned with reference sequences using MUSCLE [8].

### Genbank accession numbers

All GII.17 nucleotide sequences obtained in this study were deposited into the National Centre for Biotechnology Information (Genbank: https://www.ncbi.nlm.nih.gov/) under the accession number MN 271365-MN271367.

## Results

**Fig. 1:**
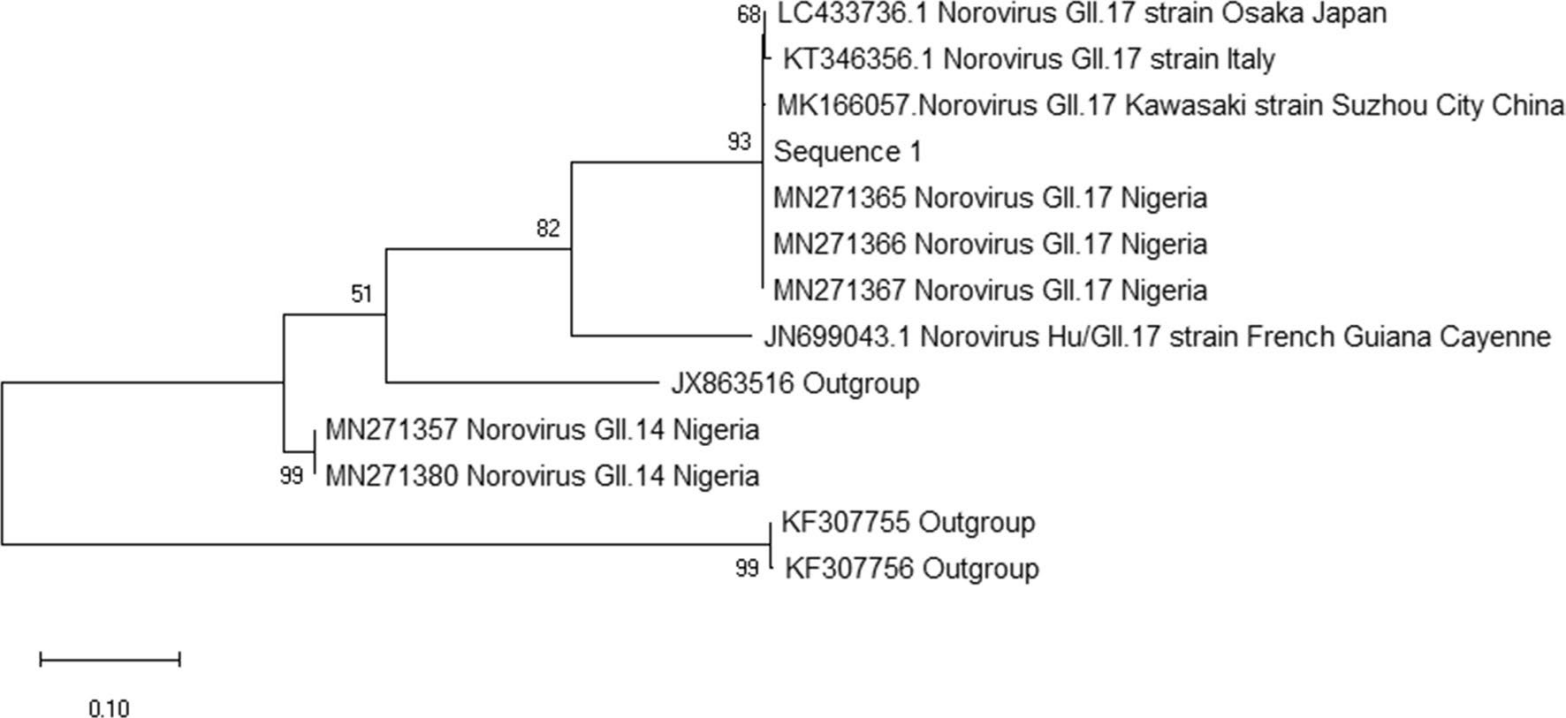
Phylogenetic tree of norovirus GII.17 isolates in this study. Norovirus GII.17 isolates among participants in this study (Accn No: MN271365-MN27136) had genetic similarity to the Kawaski GII.17 virulent recombinant strain (Accn No: MK166057).The evolutionary history was inferred by using the Maximum Likelihood method and Kimura 2-parameter model. The tree with the highest log likelihood (-6780.13) is shown. The percentage of trees in which the associated taxa clustered together is shown next to the branches. Initial tree(s) for the heuristic search were obtained by applying the Neighbor-Joining method to a matrix of pairwise distances estimated using the Maximum Composite Likelihood (MCL) approach. The tree is drawn to scale, with branch lengths measured in the number of substitutions per site. This analysis involved 13 nucleotide sequences. There were a total of 3313 positions in the final dataset. Evolutionary analyses were conducted in MEGA X

36/300 (12.0%) of study subjects were positive for norovirus. 7/36 (19.4%) of all norovirus identified where typed to be GI while others 29/30 (80.6%) belonged to norovirus GII. All norovirus was successfully sequenced and two GI genotypes were identified GI.3, 5/7 (71.4%) while the other genotype detected was GI.5, 2/7 (28.6%) Table 1. Of the GII identified, 3/29 where typed to be GII.17. The report of phylogenetic analysis shows that all GII.17 identified in this study (MN271365-MN271367) clustered with virulent Kawasaki recombinant strain (Fig. 1). Prominent illness symptoms common with all GII.17 norovirus positive patients were diarrhea, vomiting, abdominal pain and fever.

**Table 1:** Norovirus genotypes identified among children

Genotypes	Frequency (%)
GI	
GI.3	5 (71.4)
GI.5	2 (28.6)
Total	7
GII	
GII.4	22(75.9)
GII.6	2 (6.9)
GII.12	2 (6.9)
GII.14	1 (3.5)
GII.17	3 (10.3)
Total	29

## Discussion

In the present study, the frequency of detection of GI and GII norovirus was 19.4% and 80.6% respectively. To the best of our knowledge we report for the first time the detection of the less frequent GII.17 Kawasaki strain among children in South-South, Nigeria. This genotype is very virulent and has been associated with large outbreaks of gastroenteritis in Asia [9]. GII.17 norovirus strain first emerged in the winter of 2014–2015 and since then has become the predominant genotype in China and Japan [9].

It has been suggested that the capsid of GII.17 noroviruses is very stable in environmental samples [10], and therefore are capable of persisting in water bodies (surface waters, estuaries, wastewater, and Sea water and shell fish [11]. GII.17 Kawasaki has been detected in some previous waterborne norovirus outbreaks in China [12] and South Africa [13]. It is observed that over 20% of GII.17 sequences available in public data bases were collected from water related outbreaks [10]. Monitoring waste water samples and surface waters and identifying of circulating norovirus virus’s strains will be important surveillance tool for prevention and management of diarrheal illnesses in our study area.

## Limitations

Surface waters could not be screened for norovirus, this would have helped to clarify a genetic relationship between norovirus detected among study participants and prevailing strains. Sequencing of both capsid and polymerase regions of all GII.17 identified are needed to determine recombination breakpoints sites, homology model, evolutionary and phylogeographic relationships with reference GII.17 strains and recently emerging recombinant strains, this would be the work of a further study.

## Data Availability

All GII.17 nucleotide sequences obtained in this study can be accessed in genbank (Genbank: https://www.ncbi.nlm.nih.gov/) under the accession number MN 271365-MN271367.

## Abbreviations

MUSCLE: Multiple sequence comparison by log-expectation
dNTPS: Dideoxynucleotide triphosphates
RNA: Ribonucleic acid
